# The Relationship Between Recorded Bone Quality and Primary Implant Stability Measured by Insertion Torque: A Retrospective Study

**DOI:** 10.7759/cureus.112789

**Published:** 2026-07-16

**Authors:** Sameer Chauhan, Bhavan Chand Yemineni, Selva Kumar, Sanindhya Saxena, Rakhi Singh, Mazen A Almasri

**Affiliations:** 1 Department of Prosthodontics, K. M. Shah Dental College and Hospital, Sumandeep Vidyapeeth (Deemed to be University), Vadodara, IND; 2 Department of Dental and Oral Surgery, Alluri Sitarama Raju Academy of Medical Sciences, Eluru, IND; 3 Department of Oral and Maxillofacial Surgery, SRM Dental College, Chennai, IND; 4 Department of Oral and Maxillofacial Surgery, Faculty of Dental Sciences, Shree Guru Gobind Singh Tricentenary University, Gurugram, IND; 5 Department of Conservative Dentistry and Endodontics, Vananchal Dental College and Hospital, Garhwa, IND; 6 Department of Oral and Maxillofacial Surgery, Faculty of Dentistry, King Abdulaziz University, Jeddah, SAU

**Keywords:** bone density, cone-beam computed tomography, dental implants, implant stability, osseointegration

## Abstract

Background and objective

Primary implant stability is a key determinant of successful osseointegration and long-term survival. Bone density and bone quality influence the biomechanical interaction between dental implants and the surrounding bone and may affect both insertion torque and implant stability. Evaluating these parameters is important for treatment planning and predicting implant outcomes. This study aimed to investigate the relationship between cone beam (CBCT)-derived bone density and primary implant stability in patients undergoing dental implant placement.

Materials and methods

This retrospective observational study included 62 dental implants placed in 25 patients between January 2018 and December 2023. Preoperative CBCT scans were used to assess bone density at the implant sites, and bone quality was categorized into Type I, Type II, Type III, or Type IV. Primary implant stability was evaluated using insertion torque and resonance frequency analysis (RFA) and expressed as the implant stability quotient (ISQ). Statistical analyses included descriptive statistics, the Kruskal-Wallis test, the Mann-Whitney U test, and Spearman correlation analysis, with statistical significance set at p < 0.05.

Results

The mean bone density across all implant sites was 493.5 ± 274.6 HU, while the mean insertion torque and ISQ values were 30.7 ± 8.9 Ncm and 68.3 ± 7.4, respectively. Bone density differed significantly among the bone quality categories (p < 0.001), with Type I bone demonstrating the highest density values and Type IV bone the lowest. A strong positive correlation was observed between insertion torque and ISQ (ρ = 0.78, p < 0.001). Mandibular implants exhibited significantly higher ISQ values than maxillary implants (p < 0.001), whereas anterior implant sites demonstrated greater stability than posterior sites (p = 0.023).

Conclusions

Higher bone density was associated with improved primary stability and greater implant stability. A strong positive relationship was observed between insertion torque and ISQ, indicating that both measures reflect related aspects of primary implant stability. Preoperative assessment of bone density may assist clinicians in predicting implant stability and optimizing treatment planning.

## Introduction

Dental implant therapy has become a reliable and widely accepted treatment modality for replacing missing teeth, providing high long-term success rates and enhancing patient satisfaction. Successful osseointegration is influenced by several factors, with primary implant stability being recognized as one of the most critical determinants [1]. Primary stability is defined as the mechanical engagement between the implant surface and the surrounding bone at the time of placement and plays a fundamental role in minimizing micromotion during healing and promoting successful osseointegration [2]. In addition, recent advances in artificial intelligence have underscored the importance of preoperative bone assessment, with deep learning models applied to cone-beam CT (CBCT) scans demonstrating promising accuracy in predicting dental implant success and treatment outcomes [3].

Bone quality is a key determinant of primary implant stability. Variations in bone density, cortical thickness, and trabecular architecture can significantly influence the mechanical retention obtained during implant insertion [4]. Preoperative assessment of bone characteristics using CBCT has become an integral component of implant treatment planning, allowing clinicians to assess potential implant sites and predict the expected primary stability before surgery [5]. Clinically, implants placed in denser bone typically exhibit higher insertion torque values and greater initial stability compared with those placed in less dense bone [6]. Among the available methods for assessing primary implant stability, insertion torque remains one of the most commonly used clinical parameters, as it offers an immediate and objective measure of the mechanical engagement between the implant and the surrounding bone at the time of placement [7].

Insertion torque is a practical and widely used intraoperative parameter for assessing primary implant stability. It reflects the resistance encountered during implant placement and provides valuable information regarding the quality of the surrounding bone and the mechanical anchorage of the implant [7,8]. Resonance frequency analysis (RFA), expressed as the implant stability quotient (ISQ), is another commonly used method for evaluating implant stability and monitoring implant behavior during healing. Understanding the relationship between bone density, insertion torque, and implant stability may assist clinicians in treatment planning, implant selection, loading protocols, and predicting clinical outcomes.

Therefore, this study aimed to investigate the relationship between CBCT-derived bone density and primary implant stability in patients undergoing dental implant placement. The primary objective of this study was to evaluate the effect of bone density on implant stability. The secondary objectives were to assess the relationship between insertion torque and ISQ and to compare implant stability parameters across different bone quality categories and anatomical locations.

## Materials and methods

Study design and setting

This retrospective observational study was conducted at the Department of Prosthodontics, K. M. Shah Dental College and Hospital, Sumandeep Vidyapeeth (Deemed to be University), Vadodara, India, from January 2024 to March 2024. Clinical records of patients who underwent dental implant therapy between January 2018 and December 2023 were reviewed. The study protocol was approved by the Institutional Ethics Committee (SVIEC/DU/2024/Dent/jan/15 dated 14.01.2024) and conducted in accordance with the ethical principles outlined in the Declaration of Helsinki.

Sample size estimation

A priori sample size estimation was performed using G*Power software version 3.1.9.7 (Heinrich Heine University Düsseldorf, Düsseldorf, Germany). Assuming a statistical power of 80%, a significance level of 0.05, and a two-tailed correlation analysis, the minimum required sample size was calculated to be 55 implants based on a previously reported moderate-to-strong correlation (ρ = 0.50) between the ISQ and insertion torque [9]. To increase the reliability of the subgroup analyses and compensate for potential outliers, a 10% margin was added, and a target sample size of 62 implants was established. 

Patient selection

Patient records were screened according to predefined inclusion and exclusion criteria (Table 1).

**Table 1:** Inclusion and exclusion criteria for patient record screening CBCT: cone-beam computed tomography; RFA: resonance frequency analysis

Criterion type	Specific criteria/description
Inclusion criteria	Patients aged ≥ 18 years
	Underwent dental implant placement
	Complete clinical and radiographic records available
	Availability of preoperative CBCT scans
	Documented insertion torque values.
	Documented RFA measurements
	Complete implant-related information as part of the clinical record
Exclusion criteria	Incomplete documentation (missing any required clinical, radiographic, or implant-specific data)
	Uncontrolled systemic disease (e.g., uncontrolled diabetes, severe cardiovascular disease)
	Uncontrolled psychiatric disorders that could affect treatment outcomes or compliance
	Previous implant removal (failed or explanted implants at the same site)
	Implant placement in regenerated ridges (grafted bone)
	Implant placement associated with sinus augmentation procedures (e.g., sinus lift)
	Ongoing radiotherapy or chemotherapy at the time of implant treatment
	Pregnancy at the time of implant treatment

Data collection

Demographic variables, including age and sex, were obtained from patient records. Clinical variables, including implant location, dimensions, insertion torque values, stability quotient values, bone density measurements, and bone quality classification, were also recorded. To maintain confidentiality, all patient identifiers were removed before data extraction. The collected data were entered into a Microsoft Excel spreadsheet (Microsoft Corporation, Redmond, WA) and independently verified before statistical analysis.

Assessment of bone density and bone quality

Preoperative CBCT scans available in patient records were used for radiographic bone density assessment. The DICOM datasets were imported into Blue Sky Plan software (Blue Sky Bio LLC, Grayslake, IL) for image analysis. Bone density measurements were performed independently by two calibrated examiners (BCY and SS) who were not involved in implant placement, insertion torque recording, RFA, statistical analysis, or outcome assessment. Both examiners were blinded to all the clinical stability measurements and patient-related information during the evaluation process.

**Figure 1:**
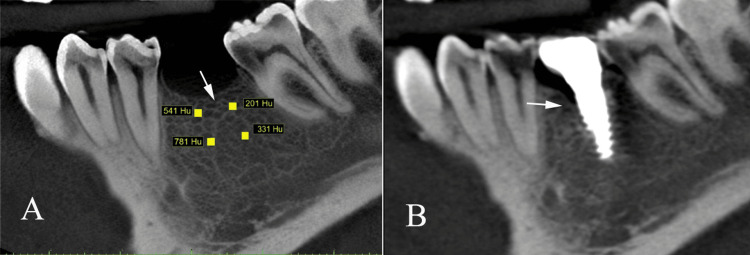
CBCT images The images show (A) preoperative measurements of bone density (HU) at the planned implant site (white arrow) and (B) the postoperative site with the implant placed (white arrow) CBCT: cone-beam computed tomography

For each implant site, the region corresponding to the planned implant location was identified on the CBCT scan, and the mean radiographic bone density value was recorded (Figure 1). Based on the radiographic density characteristics and trabecular architecture observed on CBCT images, implant sites were classified according to the Lekholm and Zarb bone quality classification as Type I (dense cortical bone), Type II (thick cortical bone surrounding dense trabecular bone), Type III (thin cortical bone with dense trabecular bone), or Type IV (thin cortical bone with sparse trabecular architecture) [5]. The final bone density and bone quality values used for the analysis were established by a consensus between the two examiners.

Reliability assessment

Both intra- and inter-examiner reliability assessments were performed to ensure the reproducibility and reliability of the CBCT-based measurements. Before the study, the two examiners underwent calibration using a set of representative CBCT scans that were not included in the final analysis. Following calibration, 20% of the study scans were randomly selected and re-evaluated after a two-week interval. For continuous variables, including bone density measurements, intra- and inter-examiner agreements were assessed using the intraclass correlation coefficient (ICC) with a two-way random-effects model and absolute agreement definition. For categorical variables, including bone quality classification, agreement was assessed using Cohen’s kappa coefficient.

Intra-examiner reliability for bone density measurements was excellent (ICC = 0.94; 95% CI: 0.89-0.97), while inter-examiner reliability was excellent (ICC = 0.91; 95% CI: 0.85-0.95). Agreement for bone quality classification demonstrated excellent reproducibility, with intra-examiner and inter-examiner Cohen’s kappa values of 0.89 and 0.86, respectively. ICC and kappa values were interpreted according to established guidelines, with values below 0.50 indicating poor agreement, 0.50-0.75 indicating moderate agreement, 0.75-0.90 indicating good agreement, and values greater than 0.90 indicating excellent agreement. Any discrepancies identified during the reassessment process were resolved through discussion and consensus between the two examiners before the final analysis.

Surgical procedure and assessment of primary implant stability

To eliminate inter-operator variability, all implant surgeries were performed by a single calibrated clinician (SC) experienced in implant dentistry. A standardized surgical protocol was followed for all the patients. Implant placement was performed using either partially or fully guided surgical templates according to the manufacturer's recommendations. The insertion torque was recorded during implant placement and used as a measure of primary implant stability. The final insertion torque recorded at complete implant seating, as documented in the clinical records, was used for statistical analysis. The insertion torque values are expressed in Ncm. The primary implant stability was further evaluated using RFA. The measurements were obtained using an Osstell Mentor device (Osstell AB, Gothenburg, Sweden).

A compatible SmartPeg transducer was attached to each implant, according to the manufacturer's instructions. Two measurements were obtained from the perpendicular directions, and the resulting ISQ values were recorded. The mean ISQ value was used for the statistical analysis. Higher insertion torque and ISQ values are considered indicative of greater primary implant stability. The implant system type, implant design, and anatomical location were also documented. Among the 62 implants included in the study, implants were placed in both the maxillary and mandibular sites and categorized according to anterior and posterior locations for subgroup analyses.

Reliability of data extraction

Both intra-examiner and inter-examiner reliability assessments were performed to ensure the validity and reproducibility of data extraction. One calibrated investigator (MAA) extracted all data from the selected records. After a two-week interval, approximately 20% of the records were randomly selected and reassessed by the same investigator to determine intra-examiner reliability. The same subset was independently evaluated by a second calibrated investigator (BCY) to assess inter-examiner reliability. Agreement between categorical variables, including bone quality classification, was evaluated using Cohen's kappa coefficient. Continuous variables including bone density measurements, insertion torque values, implant dimensions, and ISQ values were assessed using the ICC. Reliability values greater than 0.80 were considered indicative of excellent agreement. Any discrepancies identified during reassessment were resolved through discussion and consensus before final analysis.

Outcome measures

The primary outcome measures were insertion torque and ISQ values as indicators of primary implant stability. The secondary outcome measures included radiographic bone density values, bone quality classification, anatomical distribution of implant sites, and the relationship between bone density, bone quality, insertion torque, and ISQ.

Statistical analysis

Statistical analysis was performed by a blinded statistician (RS) using the statistical software (IBM SPSS Statistics for Windows, version 29.0, IBM Corp., Armonk, NY). Data were initially screened for completeness and normality. Continuous variables are expressed as mean ± standard deviation (SD) or median and interquartile range (IQR), whereas categorical variables are presented as frequencies and percentages. The Shapiro-Wilk test was used to assess the normality of continuous variables. Because the data did not follow a normal distribution, non-parametric statistical methods were used. Differences in bone density among the four Lekholm and Zarb bone quality categories were analyzed using the Kruskal-Wallis test. When significant differences were observed, Dunn's post-hoc pairwise comparisons with Bonferroni correction were performed. Comparisons of ISQ values between maxillary and mandibular implants and between anterior and posterior implant locations were conducted using the Mann-Whitney U test. The association between the insertion torque and ISQ was evaluated using Spearman's rank correlation coefficient (ρ). Effect sizes were reported as epsilon-squared (ε²) for Kruskal-Wallis analyses and rank-biserial correlation (r) for Mann-Whitney U tests. Statistical significance was established at a p-value < 0.05, with adjustment for multiple comparisons where appropriate.

Ethical considerations

Strict confidentiality of patient information was maintained throughout the study. All extracted clinical and radiographic data were anonymized and stored in password-protected electronic databases that were accessible only to the investigators. As this study involved a retrospective review of existing records, no alteration in patient management, treatment protocols, or follow-up procedures occurred as a consequence of participation in the study.

## Results

A total of 25 patients (14 females and 11 males) with a mean age of 43.0 ± 9.8 years were included in the study. The median age of the patients was 45 years (range: 28-68 years). A total of 62 implants were placed, with a mean of 2.48 ± 1.2 implants per patient. As shown in Table 1, the study included 25 patients, of whom 14 were female (56.0%) and 11 were male (44.0%). The Shapiro-Wilk test indicated a non-normal age distribution; therefore, both the mean ± SD and median values are presented.

**Table 2:** Demographic characteristics of the study population Age distribution was non‑normal (Shapiro‑Wilk p < 0.05), and hence the median and range are additionally reported SD: standard deviation

Parameter	Value
Total patients	25
Age, years	
Mean ± SD	43.0 ± 9.8
Median (range)	45 (28–68)
Sex, n (%)	
Male	11 (44.0)
Female	14 (56.0)
Implants per patient	
Mean ± SD	2.48 ± 1.2
Range	1–5
Total implants placed	62

Bone density, insertion torque, and implant stability according to anatomical location

Descriptive statistics for bone density, insertion torque, and ISQ by jaw and anatomical region are summarized in Table 3. The highest mean bone density was observed in the anterior mandible (776.0 ± 251.8 HU), followed by the posterior mandible (746.7 ± 184.4 HU). Lower bone density values were observed in the anterior maxilla (431.6 ± 142.3 HU), whereas the posterior maxilla exhibited the lowest mean bone density (193.3 ± 86.5 HU). Similarly, insertion torque values were highest in the anterior mandible (38.6 ± 7.4 Ncm), followed by the posterior mandible (36.5 ± 6.9 Ncm), whereas lower values were recorded in the anterior maxilla (28.4 ± 6.2 Ncm) and posterior maxilla (22.1 ± 5.1 Ncm). Implant stability followed a similar pattern, with the highest ISQ values recorded in the anterior mandible (75.2 ± 4.8) and the lowest values observed in the posterior maxilla (60.2 ± 6.3). The overall mean bone density, insertion torque, and ISQ values were 493.5 ± 274.6 HU, 30.7 ± 8.9 Ncm, and 68.3 ± 7.4, respectively (Table 3).

**Table 3:** Descriptive statistics of bone density, insertion torque, and ISQ according to jaw and anatomical region ISQ: implant stability quotient; SD: standard deviation

Region		N (%)	Bone density, HU, mean ± SD	Insertion torque, Ncm, mean ± SD	ISQ, mean ± SD
Maxilla	Anterior	12 (19.4)	431.6 ± 142.3	28.4 ± 6.2	68.5 ± 5.9
	Posterior	20 (32.2)	193.3 ± 86.5	22.1 ± 5.1	60.2 ± 6.3
Mandible	Anterior	8 (12.9)	776.0 ± 251.8	38.6 ± 7.4	75.2 ± 4.8
	Posterior	22 (35.5)	746.7 ± 184.4	36.5 ± 6.9	72.8 ± 5.2
Total		62 (100)	493.5 ± 274.6	30.7 ± 8.9	68.3 ± 7.4

Bone density according to bone quality classification

The relationship between bone density and bone quality classification is shown in Table 4. Statistically significant differences in bone density were observed among the four Lekholm and Zarb bone quality categories (Kruskal-Wallis H = 42.31, p < 0.001). Type I bone demonstrated the highest median bone density (985 HU), followed by Type II (712 HU), Type III (350 HU), and Type IV bone (145 HU).

**Table 4:** Comparison of bone density according to bone quality classification ^*^P < 0.05 was considered statistically significant Comparisons were performed using the Kruskal-Wallis test with Dunn’s post hoc test and Bonferroni correction IQR: interquartile range; ε²: epsilon squared effect size

Bone quality	N (%)	Bone density, HU, median (IQR)	Mean rank	Kruskal‑Wallis H statistics	P‑value	Effect size (ε²)
I (Dense cortical)	8 (12.9)	985 (895–1170)	55.8	42.31	< 0.001^*^	0.69
II (Thick cortical)	18 (29.1)	712 (660–825)	44.2			
III (Thin cortical)	26 (41.9)	350 (288–440)	27.6			
IV (Fine trabecular)	10 (16.1)	145 (112–188)	11.9			

Post-hoc pairwise comparisons (Table 5) revealed significant differences between Type I and Type III bones (p = 0.002), Type I and Type IV bones (p < 0.001), Type II and Type III bones (p = 0.006), and Type II and Type IV bones (p < 0.001). The largest effect size was observed between Type I and Type IV bones (r = 0.61), indicating a substantial difference in bone density between these categories.

**Table 5:** Post-hoc pairwise comparisons of bone density according to bone quality classification ^*^P < 0.008 was considered statistically significant Bonferroni-corrected significance level was set at α = 0.008; pairwise comparisons were performed using Dunn’s post hoc test with Bonferroni correction CI: confidence interval

Pairwise comparison of bone quality	Difference in mean ranks	Standard error	z‑statistic	95% CI for difference in mean ranks	Adjusted p‑value	Effect size (r)
I vs. II	11.6	8.42	1.38	(-4.9–28.1)	0.167	0.18
I vs. III	28.2	7.94	3.55	(12.6–43.8)	0.002^*^	0.45
I vs. IV	43.9	9.15	4.80	(26.0–61.8)	< 0.001^*^	0.61
II vs. III	16.6	6.04	2.75	(4.8–28.4)	0.006^*^	0.35
II vs. IV	32.3	7.56	4.27	(17.5–47.1)	< 0.001^*^	0.54
III vs. IV	15.7	7.05	2.23	(1.9–29.5)	0.026	0.28

Correlation between insertion torque and implant stability

The association between the insertion torque and ISQ is shown in Table 6. Spearman correlation analysis demonstrated a strong positive correlation between the ISQ and insertion torque (ρ = 0.78, 95% CI: 0.65-0.87, p < 0.001). These findings indicate that implants with higher insertion torque values generally demonstrate greater primary stability.

**Table 6:** Correlation between primary implant stability parameters ^*^P < 0.05 was considered statistically significant Correlation was assessed using Spearman’s rank correlation test ISQ: implant stability quotient; CI: confidence interval

Parameter pair	Spearman’s ρ (rho)	95% CI	P‑value
ISQ vs.iInsertion torque, Ncm	0.78	0.65–0.87	< 0.001^*^

Comparison of implant stability according to anatomical location

Comparisons of ISQ values according to anatomical location are presented in Table 7. Mandibular implants demonstrated significantly higher stability than maxillary implants, with median ISQ values of 74.0 (IQR: 70-77) and 62.5 (IQR: 58-69), respectively (U = 118.5, p < 0.001). The effect size was large (r = 0.64), indicating a clinically significant difference between the jaws. When the anterior and posterior implant sites were compared, the anterior sites demonstrated significantly greater stability than the posterior sites, with median ISQ values of 72.5 (IQR: 66-76) and 67.0 (IQR: 61-73), respectively (U = 289.5, p = 0.023). The effect size was small to moderate (r = 0.29). Overall, a higher bone density was associated with improved primary implant stability and increased insertion torque. Furthermore, a strong positive correlation was observed between insertion torque and ISQ, suggesting that both measures reflect related aspects of implant primary stability.

**Table 7:** Comparison of ISQ according to anatomical location ^*^P < 0.05 was considered statistically significant Comparisons were performed using the Mann–Whitney U test ISQ: implant stability quotient; IQR: interquartile range; U: Mann–Whitney U statistic

Comparison	Location	N (%)	ISQ, median (IQR)	Mean rank	Test statistic	P‑value	Effect size (r)
Maxilla vs. mandible	Maxilla	32 (51.6)	62.5 (58–69)	20.2	U = 118.5	< 0.001^*^	0.64
	Mandible	30 (48.4)	74.0 (70–77)	43.5			
Anterior vs. posterior	Anterior (both jaws)	20 (32.3)	72.5 (66–76)	37.0	U = 289.5	0.023^*^	0.29
	Posterior (both jaws)	42 (67.7)	67.0 (61–73)	28.4			

## Discussion

Primary implant stability is a critical prerequisite for successful osseointegration and long-term implant survival [7]. It is influenced by several factors, including bone density, bone quality, implant design, and surgical technique. This retrospective study investigated the relationship between CBCT-derived bone density and primary implant stability using insertion torque and ISQ measurements. The findings demonstrated significant differences in bone density among bone quality categories, a strong positive relationship between insertion torque and ISQ, and significantly greater implant stability at the mandibular and anterior implant sites.

The mean bone density observed in this study was 493.5 ± 274.6 HU. Significant differences in bone density were found among the four bone quality categories, with Type I bone exhibiting the highest density values and Type IV bone demonstrating the lowest values. These findings support the concept that radiographic bone density reflects the underlying differences in cortical and trabecular bone architecture. Dense cortical bone provides greater mechanical support for implant placement, whereas sparse trabecular bone offers reduced resistance to implant insertion [10]. Similar observations were reported by Turkyilmaz et al. [11], who demonstrated a positive association between bone density and implant stability in different anatomical regions. Therefore, the present findings support the clinical usefulness of preoperative CBCT assessment for characterizing implant sites and estimating expected primary stability.

A major finding of this study was the strong positive correlation between insertion torque and ISQ values (ρ = 0.78, p < 0.001). This result indicates that implants exhibiting greater resistance during insertion generally demonstrated higher stability, as measured by RFA. Although the insertion torque and ISQ assess different biomechanical characteristics of the implant-bone interface, both are influenced by the quality and quantity of the surrounding bone. Insertion torque reflects the mechanical resistance encountered during implant placement, whereas ISQ reflects the stiffness of the implant-bone complex. The strong correlation observed in the present study suggests that these measurements are closely related to indicators of primary implant stability.

The present findings are consistent with those reported by do Vale Souza et al. [9], who observed a significant positive relationship between the insertion torque and ISQ values. Similar results have also been reported by Turkyilmaz et al. [11] and Friberg et al. [8], who demonstrated that implants placed in denser bones generally achieve higher insertion torque values and superior stability measurements. Collectively, these findings reinforce the importance of bone density as a determinant of implant stability, and support the use of both insertion torque and RFA during implant treatment.

The findings of the present study are consistent with those of several previous investigations that explored the relationship between radiographic bone density and primary implant stability. Fuster-Torres et al. [12] reported significant associations between CBCT-derived bone density values, insertion torque, and resonance frequency analysis measurements, suggesting that preoperative radiographic assessment can provide valuable information regarding the expected implant stability. Similarly, Salimov et al. [13] demonstrated significant correlations between bone density values obtained from CBCT scans and both insertion torque and ISQ measurements, concluding that CBCT-based bone density assessment may be useful for predicting the initial implant stability and facilitating treatment planning.

In addition, Sennerby et al. [14] reported significant correlations between bone density measurements and primary implant stability parameters, including insertion torque and resonance frequency analysis, and suggested that bone density evaluation may serve as a useful adjunct to implant site assessment. The strong positive correlation observed in the present study between insertion torque and ISQ, together with the significant differences in bone density among the bone quality categories, further supports the clinical relevance of preoperative CBCT-based bone assessment for predicting implant stability and optimizing implant treatment protocols.

Another important observation was the significant difference in implant stability between the anatomical locations. Mandibular implants demonstrated significantly higher ISQ values than maxillary implants. This finding is consistent with the generally higher bone density observed in the mandible, particularly in the anterior region, where dense cortical bone contributes to greater mechanical engagement and improved implant stability [15]. Similar findings have been reported by Friberg et al. [8] and Sennerby and Roos [16], who noted superior primary stability in the mandibular bone compared to the maxillary bone. The present results further support the influence of anatomical location on implant stability, and highlight the importance of site-specific treatment planning.

The comparison between the anterior and posterior implant sites also revealed significantly higher ISQ values in the anterior region. This observation may be attributed to the greater cortical bone thickness and higher bone density typically present in the anterior than in the posterior regions. Previous investigations have similarly reported improved stability outcomes at anterior implant sites, particularly in the anterior mandible, where dense cortical bone contributes to enhanced implant anchorage [17,18]. Meredith [19] suggested that implant stability is influenced not only by bone characteristics but also by implant design, thread geometry, surgical technique, and operator experience. Advances in implant surface technology and macrodesign have enabled acceptable primary stability even at sites with relatively low bone density. Nevertheless, most evidence indicates that bone density remains one of the most influential factors affecting primary implant stability.

Several limitations of this study should be considered when interpreting its findings. First, the retrospective design limited control over potential confounding variables and relied on the accuracy of existing clinical records. Second, the sample size was relatively modest and was obtained from a single institution, which may limit the generalizability of the findings. Third, bone density measurements were derived from CBCT datasets, which may be influenced by imaging parameters and software-based calibration. Finally, only primary stability measurements obtained at the time of implant placement were evaluated, and long-term clinical outcomes were not assessed.

Despite these limitations, the present study provides clinically relevant insights into the association between radiographic bone density and primary implant stability in routine implant practice. However, the findings should be interpreted with caution because CBCT-derived radiographic density values are not directly equivalent to medical CT Hounsfield units; implant-related variables, such as the implant system and macro-design, were not evaluated in the statistical analyses; and multiple implants from the same patient were analyzed as independent observations without adjustment for potential intra-patient clustering. Consequently, the reported associations should be considered exploratory within the context of the study design. Nevertheless, the observed relationship between insertion torque and ISQ supports the complementary use of these measures in the assessment of primary implant stability. Future prospective multicenter studies with standardized CBCT calibration protocols, comprehensive reporting of implant-related variables, and statistical models accounting for patient-level clustering are warranted to validate and extend these findings.

## Conclusions

Within the limitations of this retrospective study, the CBCT-derived bone density was significantly associated with primary implant stability. Significant differences in bone density were observed among the bone quality categories, and implants placed in denser bones exhibited more favorable stability characteristics. A strong positive correlation was identified between insertion torque and ISQ, with insertion torque serving as a significant predictor of implant stability. These findings suggest that the preoperative assessment of bone density may assist clinicians in anticipating primary implant stability and optimizing implant treatment planning, surgical protocols, and loading strategies.
